# The hepatitis C cascade of care in people who inject drugs in Dar es Salaam, Tanzania

**DOI:** 10.1111/jvh.12966

**Published:** 2018-07-30

**Authors:** Zameer Mohamed, John Rwegasha, Jin U. Kim, Yusuke Shimakawa, Lila Poiteau, Stéphane Chevaliez, Sanjay Bhagani, Simon D. Taylor‐Robinson, Mark R. Thursz, Jessie Mbwambo, Maud Lemoine

**Affiliations:** ^1^ Department of Hepatology Imperial College London St Mary's Hospital London UK; ^2^ Department of Gastroenterology Muhimbili National Hospital Dar es Salaam Tanzania; ^3^ Unité d'Épidémiologie des Maladies Émergentes Institut Pasteur Paris France; ^4^ Department of Virology French National Reference Center for Viral Hepatitis B, C and Delta Hopital Henri Mondor Université Paris‐Est Créteil France; ^5^ The Royal Free Hospital London UK; ^6^ Department of Psychiatry Muhimbili University of Health and Allied Sciences Muhimbili National Hospital Dar es Salaam Tanzania

**Keywords:** cascade of care, fibrosis, hepatitis C virus, people who inject drugs, Sub‐Saharan Africa

## Abstract

The World Health Organisation has recently called for hepatitis C virus (HCV) elimination and has identified people who inject drugs (PWID) as a key population to scale‐up screening and linkage to care. This study reports the cascade of care for HCV in PWID attending the largest opioid substitution treatment (OST) clinic in Dar‐es‐Salaam, Tanzania. Between February 2011 and March 2016, HCV serology for all PWID registered at the Muhimbili National Hospital OST clinic, Dar‐es‐Salaam were obtained from records. In 2015, consecutive HCV‐seropositive PWID were invited to undergo a clinical evaluation including epidemiological questionnaire, liver stiffness measurement (Fibroscan) and virological analysis (HCV RNA viral load and genotyping). During the study period, 1350 persons registered at the OST clinic: all had a HCV serology including 409 (30%) positive results. Among the HCV‐seropositive individuals, 207 (51%) were active attenders and 153 (37%) were enrolled for clinical assessment: 141 (92%) were male, median age: 38 years (IQR 34‐41), and 65 (44%) were co‐infected with HIV; 116 patients (76%) had detectable HCV RNA, with genotypes 1a (68%) and 4a (32%); 21 (17%) had clinically significant fibrosis (≥F2) and 6 (5%) had cirrhosis (F4). None were offered HCV treatment. Chronic hepatitis C among PWID enrolled in the OST centre in Dar‐es‐Salaam is frequent, but its continuum of care is insufficient; integration of HCV diagnosis and treatment should form a part of OST intervention in PWID in Tanzania.

**Abbreviations**
ALT: alanine aminotransferase
APRI: aspartate platelet ratio index
AST: aspartate transferase
AUROC: area under the receiver operating characteristic curve
BBV: blood borne virus screening
BR: bilirubin
DAA: direct‐acting antiviral
DBS: dry blood spot
GGT: gamma‐glutamyl transferase
GPR: gamma‐glutamyl transferase to platelet ratio
HBV: hepatitis B virus
HCV: hepatitis C virus
HCVcAg: HCV core antigen quantification
LSM: liver stiffness measurement
NAT: nucleic acid testing
NSP: needle‐and‐syringe‐programmes
OST: opioid substitution treatment
PEPFAR: President's emergency plan for AIDS relief
PWID: people who inject drugs
UNODC: United Nations Office on Drugs and Crime

## 1 INTRODUCTION

An estimated 71 million people are chronically infected with the hepatitis C virus (HCV) worldwide and each year between 400 000 and 700 000 deaths are attributable to this virus.^1^, ^2^ The World Health Organisation (WHO) has recently called for HCV elimination.^3^ In its 2016‐2021 global hepatitis plan, the WHO defined ambitious strategies to achieve 90% reduction in new HCV cases and 65% decrease in HCV‐related mortality by 2030.^3^ Intravenous drug use is a major driver of HCV spread worldwide and the WHO has clearly identified people who inject drugs (PWID) as a key population to target for HCV screening, prevention and care.^4^ It is estimated that 10 million of 16 million PWID across the world are positive for HCV antibody.^5^

In sub‐Saharan African (SSA), the use of injectable drugs has been long considered as a minor issue in terms of the numbers of people involved. Globally, 8% of PWID are estimated to reside in sub‐Saharan Africa. A recent influx of heroin has led to a rise in injecting drug use, which has been recognized as a growing concern in coastal East Africa. In particular, Kenya, Tanzania, Madagascar, Reunion, Seychelles and Mauritius have endured a sharp increase in local heroin use. Heroin trafficking follows the southern pathway, a network of mainly maritime routes originating from Afghanistan and Pakistan, traversing the Indian Ocean, before arriving in East Africa.^6^ Hence, East Africa is a point of geographical significance and is widely considered the gateway for African narco‐trafficking, with 90% of the imported heroin destined for distribution to both continental and global sites. In 2013, it was estimated that 22 tons of heroin passed through East Africa annually, with 2.5 tons being consumed locally.^7^ Costing as little as USD$1 per “hit,” it is estimated that 30 000‐45 000 people who inject heroin in Tanzania.^8^

Data on the burden of HCV among PWID in SSA are scarce. A study in Sénégal reported a high prevalence of HCV infection in drug users, but did not analyse the severity of the liver disease and furthermore, the drug users were mainly noninjectors.^9^ In East Africa, there have been a handful of descriptive studies, restricted to assessing the seroprevalence of HCV infection, ranging from 28%‐53% in Tanzania to 42% in Kenya.^10^, ^11^, ^12^ Lack of access to HCV nucleic acid testing (NAT), is a major obstacle to identifying chronically‐infected individuals encountered across resource‐limited settings.^13^

Similarly, evaluating the stage of liver disease is problematic. The WHO 2016 HCV guidelines advocate the use of simple noninvasive scores to stratify liver disease (aspartate transferase [AST] to platelet ratio index [APRI] and Fib‐4) in the absence of ready access to liver biopsy or Fibroscan.^4^ In Africa, in particular in SSA, there has been limited work done to evaluate the performance of noninvasive scores of fibrosis in patients with HCV. Bonnard et al^14^ demonstrated a modest performance of APRI and Fib‐4 to predict fibrosis level in HCV genotype 4 infected patients in Egypt, while another study found good performance of gamma‐glutamyl transferase to platelet ratio (GPR) in the same population.^15^ Moreover, the severity of liver disease and cascade of care among HCV‐infected PWID has been poorly documented in Africa.

This study aimed to assess the severity of liver disease and cascade of care for HCV‐infected PWID registered at one of the largest opioid substitution therapy (OST) clinic in Dar‐es‐Salaam, Tanzania, East Africa. We also determined the proportion of significant liver fibrosis or cirrhosis using liver stiffness measurement (LSM), as measured by ultrasound‐based transient elastography, and assessed the diagnostic accuracy of APRI, Fib‐4 and GPR using LSM as a reference test.

## 2 METHODS

### 2.1 Study population

Between February 2011 and March 2016, routine blood borne virus screening (BBV) was offered to all PWID registering at the largest OST clinic in Tanzania, located at Muhimbili National Hospital (MNH), Dar‐es‐Salaam. Between April and July 2015 all those with known positive HCV serology were invited to participate in further evaluation, undergoing a clinical review including a comprehensive liver assessment, including fasting LSM, abdominal ultrasound, and the following blood tests: liver function tests, full blood count and dry blood spot (DBS) sampling. All participants with a positive HCV serology had an assessment of HCV RNA level and genotyping. For the purpose of this study, participating in the clinical assessment was considered as linkage to care.

Demographic (age, gender), lifestyle (smoking and drug habit, alcohol consumption), age of first injection of drug, history of hepatitis B virus (HBV) and/or HIV infections (including most recent CD4 count (when available), knowledge of HCV status and clinical data (medication and medical history) were collected at time of enrolment and recorded on standardized forms.

### 2.2 Ethical consideration

The study received clearance from both Muhumibili University for Health and Allied Sciences (MUHAS) and the Tanzanian National Institute for Medical Research (NIMR) (NIMR/HQ/R8a/Vol.ix/2298) institutional review board panels. Participants were enrolled in the study after providing written consent.

### 2.3 Assessment of liver fibrosis:

#### 2.3.1 APRI, Fib‐4 and GPR fibrosis scores

The following blood parameters were measured at the study enrolment: AST, alanine aminotransferase (ALT), gamma‐glutamyl transferase (GGT), bilirubin (BR) and platelet count. These tests were used to calculate APRI (AST (IU/L)/its upper limit of normal (ULN)/platelet count (10^9^/L) × 100), Fib‐4 (age (years) × AST (IU/L)/(platelet count [10^9^/L] x √ALT [IU/L]) and GPR (GGT (IU/L)/its upper limit of normal (ULN)/platelet count (10^9^/L × 100) as markers of liver fibrosis. Cut‐off values were determined, as previously recommended.^4^, ^16^, ^17^

#### 2.3.2 Transient elastography

LSM was obtained in fasting patients using ultrasound‐based transient elastography (Fibroscan^®^ 402, Echosens, Paris, France) and was performed by trained experienced operators according to the manufacturer's protocol.^18^ Results were expressed in kilopascal (kPa) as the median value of 10 successful acquisitions. Failure was defined as no single successful measurement (valid shot = 0) and unreliable measurement was defined as IQR/LSM of >0.30 when LSM is ≥7.1 kPa.^19^

To estimate fibrosis stage, we used the following cut‐off values of Fibroscan^®^ in accordance with the WHO 2016 guidelines for HCV screening, care and treatment; ≥7.0 kPa for ≥F2 disease and ≥11.0 kPa for ≥F4 disease, respectively.^4^

### 2.4 Serological methods used to detect anti‐HCV, anti‐HIV Abs and HBsAg

Serology testing for anti‐HCV, anti‐HIV Abs and HBsAg were performed using the Abbott AXSYM^®^ system (Abbott Diagnostics, Chicago, Illinois) according to the manufacturer's instructions, at the MNH clinical chemistry department, Dar‐es‐Salaam.

### 2.5 HCV RNA detection and quantification

HCV RNA was quantified using the Cobas Ampliprep/Cobas TaqMan HCV version 2 (CAP/CTM; Roche Molecular Systems Pleasanton, California), real‐time polymerase chain reaction (RT‐PCR) assay according to the manufacturer's instructions.

### 2.6 HCV genotyping

Samples were analysed at Henri‐Mondor Hospital (Creteil, France) for genotyping and HCV RNA confirmation. HCV genotypes and subtypes were determined by means of the reference method that is sequencing of the nonstructural 5B region of the HCV genome, followed by phylogenetic analysis, as previously described.^20^

### 2.7 Statistical analysis

The characteristics of the study participants were presented by median and interquartile range for the continuous variables and percentage for categorical variables. Factors associated with clinically significant fibrosis (≥F2), determined by Fibroscan^®^, were identified in patients with chronic HCV infection (ie, anti‐HCV positive and HCV RNA positive) using a univariable logistic regression. The variables below were assessed: age; sex; alcohol intake; BMI; co‐infection with HIV or HBV; untreated HIV; HCV RNA levels in serum; and HCV genotype. The statistical significance was determined as a two‐sided *P*‐value of <0.05.

The diagnostic performance of APRI, Fib‐4 and GPR to indicate clinically significant fibrosis (≥F2) or cirrhosis (F4), determined by Fibroscan^®^, was assessed using the area under the receiver operating characteristic curve (AUROC). By applying the previously validated cut‐off values of APRI, Fib‐4 and GPR, the sensitivity and specificity were obtained.

## 3 RESULTS

### 3.1 Hepatitis C cascade of care

**Figure 1:**
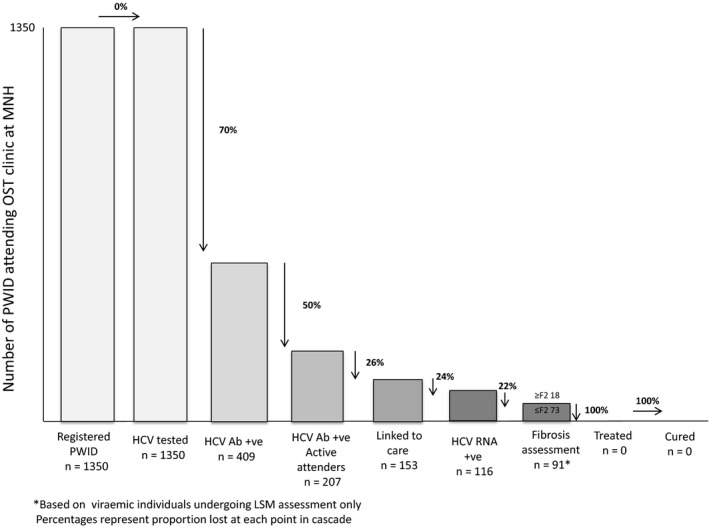
HCV cascade of care for patients attending the OST centre at MNH

A total of 1350 PWID were registered at the OST clinic at MNH since its inception. All were offered and accepted to have screening for anti‐HCV antibody, 409 (30%) tested positive, of whom 207 (51%) were active attenders, 131 (32%) had defaulted, 49 (12%) had died, 22 (5%) had been successfully discharged from the programme. As part of this research project between April and July 2015, 153 (74%) of consecutive active HCV‐seropositive attenders were successfully linked to further clinical and virological evaluation. 116 (76%) were deemed to be HCV RNA positive and 21 (17%) were suspected to have significant fibrosis on the basis of high LSM values. None had access to any anti‐HCV therapy. Figure 1 summarizes the cascade of care for HCV in this population.

### 3.2 General characteristics

During the study timeframe, 153 consecutive PWID (median age 38 [IQR 34‐41, male sex 141 [92%]) with positive anti‐HCV antibody were enrolled, of whom 116 (76%; 95% CI 68%‐82%) were HCV RNA positive. The median viral load was 5.7 Log IU/mL (IQR 4.0‐6.3 Log IU/mL) and genotypes 1a (75, 68%) and 4a (35, 32%) were the only genotypes identified. 79 (52%) PWID reported to having had a history of needle sharing, with 66 (62%) aware of their HCV status. 64 (42%) had abstained from drinking alcohol as part of their rehabilitation, with only 24 (16%) continuing to drink excessively. 15 PWID (10%) were co‐infected with HBV and 65 (43%) were co‐infected with HIV with a median CD4 cell count of 553/mm^3^ (IQR 187‐769), of whom 41 (63%) were on antiretroviral therapy. The main characteristics of the study population are presented in Table 1.

**Table 1:** Clinical characteristics of HCV antibody positive patients undergoing clinical assessment

	Recruited (n = 153)
Median age, years (IQR)	38 (34‐41)
Male sex (%)	141 (92)
Current history of heavy alcohol use (%) (defined as >40 g intake per day)	24 (16)
Needle sharing practice (%)	79 (52)
Knowledge of HCV status (%) (n = 107)	66 (62)
Median time from starting IVDU, years (IQR)	22 (18‐26)
Alcohol abstinence since enrolment (%)	64 (42)
Median BMI, kg/m^2^ (IQR)	20.8 (19‐23)
Anti‐HIV positive (%)	65 (44)
CD4 count, cells/mm^3^ (IQR)	553 (187‐769)
On ART (%)	41 (27)
Tenofovir/Emtricitabine/Efavirenz (%)	22 (54)
Tenofovir/Lamivudine/Efavirenz (%)	10 (24)
Zidovudine/Lamivudine/Efavirenz (%)	9 (22)
HBsAg positive (%)	15 (10)
Median ALT, IU/L (IQR)	30 (20‐49)
Median AST IU/L (IQR)	39 (29‐55)
Median GGT, IU/L (IQR)	58 (31‐110)
Median total bilirubin, μmol/l (IQR)	7 (5‐10)
Median platelet count, x10^9^/l (IQR)	198 (158‐250)
Median APRI (IQR)	0.59 (0.38‐0.92)
Proportion of patients with APRI ≥ 2, n (%)	7/126 (6)
Median Fib‐4 (IQR)	1.36 (0.92‐2.01)
Median GPR (IQR)	0.32 (0.20‐0.86)
Median LSM (IQR)	5.4 (4.4‐6.5)
Distribution of fibrosis stage according to LSM (%)	
F0‐1	(102, (83))
F2‐3	(15, (12))
F4	(6, (5))
Positive HCV RNA (%)	116 (76)
Genotype (n = 110)	
1a (%)	75 (68)
4a (%)	35 (22)
Median HCV RNA (log IU/mL), (n = 116)	5.7 (4.0‐6.3)

ART, antiretroviral therapy; LSM, liver stiffness measurement.

### 3.3 Severity of liver fibrosis

The median noninvasive scores of liver fibrosis are indicated in Table 1. 122 of 123 (99%) patients had valid LSM measurements (median LSM value 5.4 kPa [IQR 4‐4‐6.5]); 15 (12%) had LSM values suggesting clinically significant or extensive liver fibrosis (F2‐F3) and 6 (5%) patients were classified as cirrhotic (F4), none had decompensated cirrhosis. In patients with positive HCV RNA, univariable analysis did not identify any factors associated with clinically significant liver disease ([≥F2] based on LSM values).

### 3.4 Performance of biochemical scores using LSM as a reference

The performance of each biochemical score for correctly categorizing significant liver fibrosis or cirrhosis, using LSM as a reference, is summarized in Table 2. With the AUROC being below 0.60, the overall performance of these tests for the diagnosis of fibrosis and cirrhosis was not acceptable. To diagnose of clinically significant fibrosis (≥F2), the low cut‐off APRI (<0.5) had the best sensitivity (73%), while a cut‐off value at 2 had a poor sensitivity for the diagnosis of cirrhosis, but a very good specificity (95%). However, its utility is hampered by the fact that more than half of the resulting scores presided in the “grey zone,” between the different cut‐off values.

**Table 2:** Diagnostic accuracy of APRI, Fib‐4 and GPR using LSM as a reference in patients with chronic hepatitis C

	F0‐1 vs F2‐4		F0‐3 vs F4	
APRI				
AUROC (95% CI)	0.58 (0.41‐0.75)		0.51 (0.06‐0.97)	
Cut‐off values	0.5	1.5	1.0	2.0
Sensitivity/specificity (%)	73/34	27/88	50/77	25/95
Correctly classified (%)	41	77	76	92
PPV/NPV (%)	20/85	33/84	10/97	20/96
Positive/negative LR	1.1/0.8	2.2/0.8	2.2/0.7	4.9/0.8
Fib‐4				
AUROC (95% CI)	0.51 (0.34‐0.68)		0.48 (0.09‐0.87)	
Cut‐off values	1.45		3.25	
Sensitivity/specificity (%)	43/50		14/92	
Correctly classified (%)	49		78	
PPV/NPV (%)	16/80		29/83	
Positive/negative LR	0.9/1.1		1.8/0.9	
GPR				
AUROC (95% CI)	0.56 (0.37‐0.75)		0.62 (0.34‐0.90)	
Cut‐off values	0.28		0.28	
Sensitivity/specificity (%)	69/40		75/39	
Correctly classified (%)	45		41	
PPV/NPV (%)	19/87		6/97	
Positive/negative LR	1.2/0.8		1.2/0.6	

AUROC, area under the receiving operator curve; PPV, positive predictive value; NPV, negative predictive value.

## 4 DISCUSSION

The diagnosis of HCV among PWID in Tanzania accessing the largest OST clinic is limited to serology testing on registration. Our study revealed that about a third had prior exposure to HCV, of whom half are regular attenders at the OST clinic. Of those not engaging in the clinic, 12% of patients are known to have died. In addition, close to a third of patients are reported as “defaulters.” With the clinical outcomes not known for this group, it is conceivable that the true mortality rate could be higher.

Clinical evaluation from a sub‐group of consecutive HCV‐seropositive PWID receiving OST, found that nearly half were co‐infected with HIV and three‐quarters were HCV viraemic, with genotypes 1a and 4a being identified. This is in line with the only other report of genotyping performed in PWID in East Africa (Kenya).^21^ In addition, we found 17% of patients with significant liver fibrosis according to LSM including 5% with cirrhosis. At the time of writing, there has been no access to direct‐acting antiviral (DAA) interferon‐free therapy for PWID in Tanzania.

To our knowledge, this is the first study to provide a comprehensive screening and assessment cascade of care for HCV in PWID in Africa. There have been two previous studies estimating HCV seroprevalence among PWID in Tanzania, reporting a prevalence of 28% and 53%, respectively.^10^, ^12^ Both previous studies also confer our finding of a heavily predominant male population.^10^, ^12^ Although this may truly reflect that injecting drug use is more commonly practiced among men, the difference is likely to be exaggerated as women may represent a more marginalized and stigmatized proportion of this under‐served population. According to the United Nations Office on Drugs and Crime (UNODC) world drug report 2015, it is estimated that 1‐in‐5 users of illicit substances across the globe are women, however, currently on average only 1‐in‐5 PWID entering rehabilitating programmes are female.^22^

Despite offering systematic BBV screening at registration and the high prevalence of HCV, the OST centres in Tanzania do not offer linkage to care for any cause of chronic viral hepatitis. In addition, no repeat testing is offered after registration, despite the on‐going risk of transmission. Yet, BBV counselling represents an important opportunity to educate and promote behavioural modification of high‐risk practices among PWID. Although the impact of knowing HCV infection status on high‐risk practice is conflicting, PWID awareness of HCV status is universally reported as poor (32%).^23^, ^24^ This is in contrast to attenders at the OST clinic we describe, where 62% of patients were aware of their HCV status. Thus, it is conceivable that access to HCV care and treatment, in addition to counselling, would strengthen the provision of healthcare and incentivize increased retention across the cascade of care. It will be important to investigate how this can be best implemented in a resource‐limited OST setting.

An important concept in improving the cascade of care for HCV in this setting is the simplification of the screening and linkage to care process. Traditionally, screening involves a HCV antibody test (exposure), followed by a second test to confirm HCV RNA (active infection), which is not only expensive, but it is also difficult to access in resource‐limited settings. A number of innovative solutions are available to condense the diagnostic algorithm and improve uptake of testing. First, HCV core antigen quantification (HCVcAg), which is a surrogate measure of HCV RNA, has a relatively low cost (at US$10 per test),^25^ with a fully automated assay available on the Abbott ARCHITECT^®^ platform. It has been recently validated in African resource‐limited settings and has been proposed as a one‐step diagnostic alternative to conventional screening in such an environment.^26^, ^27^, ^28^ An additional challenge to testing PWID is poor venous access. To overcome this issue, Grebley and colleagues have demonstrated good performance of using blood drawn from a finger‐prick on the GeneXpert^®^ HCV RNA assay, which also has relatively low cost (at US$17 per test).^29^ Alternatively, we have recently developed a diagnostic algorithm for resource‐constraint PWID settings, which incorporates HCVcAg in serum and dry blood spots (DBS).^28^ Integrating decentralized testing in existing needle‐and‐syringe‐programmes (NSP) in Dar‐es‐Salaam, such as the one run by the nongovernmental organization “Médecins Du Monde,” may provide an excellent opportunity to re‐engage those who have been lost to follow‐up. Finally, with consideration that the predominant genotypes in PWID in East Africa are 1a and 4a and the existence of DAAs offering pan‐genotypic activity, some of which are now accessible as generic treatments (eg sofosbuvir plus daclatasvir/ledipasvir), we propose genotyping as nonessential to the diagnostic work‐up.

In addition to solving the HCV diagnostic challenges, the advent of noninvasive measures of liver fibrosis have provided a viable solution to identifying patients urgently in need of treatment; however, their utility in resource‐limited settings has been poorly documented. To our best knowledge, our study is the first to report the burden of liver fibrosis among PWID in SSA, with 17% of patients having clinically significant fibrosis (≥F2), including 5% with cirrhosis according to LSM. This is lower than the rate reported in European, Australian and American cohorts of PWID with chronic HCV, where the proportion of subjects with significant fibrosis is often over 30%.^30^, ^31^, ^32^ This difference can be attributed to; (a) the relatively young age (median age 38 years) of our study population, (b) a small proportion of patients with excessive alcohol consumption (16% in our study), (c) a reduced contribution from HIV co‐infection, owing to good coverage of antiretroviral therapy (64%), reflected by a relatively preserved CD4 count (553 cells/mm^3^), (d) the low BMI and (e) the African ethnicity, which might play a protective role in liver fibrogenesis.^33^

Despite the potential utility of Fibroscan^®^ to determine stage of liver fibrosis, its cost prohibits its wide scale use resource‐limited settings. The WHO has advocated the use of biochemical fibrosis markers to identify patients with advanced liver disease in settings where access to liver biopsy or Fibroscan^®^ is limited.^4^ Our setting is a typical example where such simple noninvasive staging tools would be valuable. However, in our study, there was a surprisingly poor concordance of such biochemical markers with the LSM, with the majority of scores deemed inconclusive. Although this inference is limited by the lack of statistical power due to the relatively small number of patients with F2‐F4 fibrosis, it is important to note that serum biochemistry and platelet count can be influenced by alcohol consumption and infections (particularly HIV), which are both well‐documented issues in the PWID community.^34^ The latter is an issue which has also been identified in a recent validation of APRI in HCV/HIV co‐infection in Cambodia.^35^

Mathematical models have shown that scaling‐up harm reduction services, through OST and NSPs, in combination with DAA therapy will help achieving HCV elimination in PWID.^36^ In populations with escalating incidence of PWID, a failure to integrate harm reduction strategies into any proposed healthcare intervention can have a significant impact on HCV transmission, as evidenced by the recent “opioid crisis” in parts of North America.^37^, ^38^ In Tanzania, there are an estimated 50 000 PWID, with only 3000 engaging in a local OST programme.^10^ Despite the existence of NSP in Dar‐es‐Salaam,^39^ more than half of PWID surveyed admitted to a history of needle sharing. Thus, priority should be reserved for escalating harm reduction and optimizing the diagnostic and assessment pathway in anticipation for improved access to DAA therapy.

With the exception of Egypt, there has been very limited experience of treating HCV with DAAs in Africa. One of the major barriers to scaling‐up access to DAA therapy is the cost. This is particularly the case in SSA, where the majority of healthcare costs are funded personally. With 48% of Tanzanians living below the international poverty line (<USD $1.90), even the discounted cost of 28 days treatment ($300) is unacceptable.^40^ More recently, low‐cost generic DAAs are being manufactured in countries such as Egypt, Morocco and India, with the cost of 28 days of Sofosbuvir falling to USD$15.^41^ Furthermore, the WHO has endorsed the use of generic treatments, having recently pre‐qualified generic Sofosbuvir and adding various combinations to the WHO essential medications list.^42^

It is important to recognize the role of local policymakers in taking responsibility for the implementation of a sustained test‐and‐treat policy. The commitment to a national action plan in Tanzania ensures that there is an agreed personnel and financial strategy in place to achieve the prescribed objectives. In 2013, only 63% of WHO member states (including Tanzania) had not submitted a national hepatitis strategy.^43^ Although Tanzania is considered a pioneer in Africa for its OST programme, it is dependent on funding through the President's emergency plan for AIDS relief (PEPFAR). Recent changes to the local healthcare agenda threaten to deprioritize the OST programme, while the well‐publicised reduction of international foreign aid also poses a real risk to the existence of this once‐acclaimed initiative.

Our study has some limitations: first, it reflects HCV‐related liver disease among PWID compliant with the OST programme and conclusions cannot be extended to those not engaging or lost to follow‐up. This may have introduced a selection bias, as the most “healthy” PWID are enrolled in OST centres. Thus, the proportion of patients with severe liver disease has been certainly underestimated. Secondly, the lack of access to HIV viral load and sporadic CD4 cell count measurement limited the understanding of the severity of HIV infection. This may have impacted assessment of the role of HIV infection in the severity of the liver disease. Lastly, we used LSM and not liver biopsy as a gold standard to assess the performance of biochemical markers of fibrosis, as liver biopsy is practically difficult to perform in Africa, in particular in PWID.

In conclusion, injecting drug use is an underappreciated problem in SSA, with a significant proportion of PWID who are chronically infected with HCV currently unable to access curative therapy. The existing OST and NSP provide a valuable opportunity to optimize the screening, assessment and linkage to HCV care. However, there is urgent need for a low‐cost simplified strategy to improve access to diagnostics and prioritization of patients for curative HCV treatment in the resource‐limited PWID setting. Ultimately, commitment of healthcare policymakers to invest in scaled‐up coverage of OST and NSP, incorporating HCV screening and treatment, have become urgent priorities to achieve the WHO hepatitis elimination objectives.

## CONFLICT OF INTEREST

All authors have no conflict of interest to declare related to this study.
